# Neuroprotective effects of noble gases following acquired brain injury: a narrative review

**DOI:** 10.3389/fphar.2026.1908611

**Published:** 2026-07-24

**Authors:** Hongru Li, Zeming Xie, Sicen Wan, Gang Chen, Jiahe Wang, Xiang Li

**Affiliations:** Department of Neurosurgery, The First Affiliated Hospital of Soochow University, Suzhou, Jiangsu, China

**Keywords:** acquired brain injury, argon, helium, neuroprotection, noble gas, xenon

## Abstract

Noble gases were chemically inert elements that have attracted increasing interest because of their potential neuroprotective properties. Among them, xenon had long been used as an inhalational anesthetic, while xenon, argon, and helium had also been investigated for possible neuroprotective effects. This narrative review focused on acquired brain injury, particularly ischemic stroke, post-cardiac arrest hypoxic-ischemic brain injury, and traumatic brain injury. Preclinical studies had suggested that several noble gases might reduce secondary neuronal injury, although the strength and consistency of evidence varied across gases and experimental models. Proposed mechanisms included attenuation of excitotoxicity, apoptosis, oxidative stress, and neuroinflammation, although these pathways had not been fully clarified for all gases. This review summarizes and compares the available preclinical and early clinical evidence on xenon, argon, and helium in acquired brain injury. Among these gases, xenon currently had the strongest and most consistent evidence base, whereas evidence for argon remained heterogeneous and evidence for helium was limited. Although the preclinical findings were promising, clinical translation remains limited, and further standardized studies were needed to define efficacy, safety, timing of administration, and feasible delivery strategies.

## Introduction

The noble gases, including helium, neon, argon, krypton, xenon, and radon, were chemically inert elements whose closed-shell electronic structure made them largely nonreactive under physiological conditions. Among them, xenon had been studied most extensively and had been used clinically as an inhalational anesthetic (Cullen and Gross, 1951). Beyond its anesthetic properties, xenon had attracted increasing attention because of its potential neuroprotective effects. In addition to xenon, argon and helium had also been investigated for possible neuroprotective properties in experimental models (Coburn et al., 2008; Koziakova et al., 2019; Chen et al., 2026; Ezzeddine, 2011). Other noble gases, such as krypton and neon, had also been explored, but the currently available evidence remained limited and lied outside the main scope of this review (Koziakova et al., 2019; Antonova et al., 2024; Pagel et al., 2007). Therefore, this narrative review focused on the neuroprotective effects of xenon, argon, and helium in acquired brain injury.

Acquired brain injury was a broad term that generally referred to brain damage occurring after birth as a result of traumatic or non-traumatic causes. This review focused primarily on traumatic brain injury, ischemic stroke, and post-cardiac arrest hypoxic-ischemic brain injury, which together represented major forms of acquired brain injury examined in both experimental and early clinical studies. Among the major forms of acquired brain injury, traumatic brain injury (TBI) remained an important cause of death and disability worldwide (Capizzi et al., 2020). In China, the absolute number of TBI cases remains substantial, and TBI was reported more frequently in men than in women (Jiang et al., 2019). The leading cause of TBI also varied across regions. In China, common causes of TBI included traffic accidents, falls, and violence (Capizzi et al., 2020).

Neuroprotection referred to strategies that preserved neuronal structure and function and limit secondary injury after brain insult (Vajda, 2002). In acquired brain injury, secondary injury mechanisms—including excitotoxicity, oxidative stress, inflammation, apoptosis, and blood-brain barrier disruption—substantially contributed to neurological deterioration (Lee, 2021). Accordingly, therapies capable of attenuating these secondary processes might improve histological and functional outcomes after brain injury (Lee, 2021).

Against this background, noble gases—particularly xenon, and to a lesser extent argon and helium—had emerged as potential neuroprotective agents in experimental models of acquired brain injury (Shin et al., 2021; Liang et al., 2022; Magliocca and Fries, 2021). However, their translation into clinical practice remained limited because the available evidence was still largely preclinical, and practical issues such as cost, gas delivery, and the need for specialized circulation systems remain important barriers (Neice and Zornow, 2016; Merigo and Ristagno, 2026).

This narrative review summarized and compared the available preclinical and early clinical evidence on xenon, argon, and helium in acquired brain injury, with a particular focus on traumatic brain injury, ischemic stroke, and post-cardiac arrest hypoxic-ischemic brain injury. The study also discussed proposed mechanisms, major limitations of the current literature, and challenges for future clinical translation.

This review focuses on traumatic brain injury (TBI), ischemic stroke, and post-cardiac arrest hypoxic-ischemic brain injury. The following abbreviations are used throughout this article: middle cerebral artery occlusion (MCAO), including transient (tMCAO), permanent (pMCAO), and embolic (eMCAO) variants; cardiopulmonary resuscitation (CPR); recombinant tissue plasminogen activator (rt-PA); and mild therapeutic hypothermia (MTH).

## Search strategy

A literature search was conducted in PubMed, Embase, the Cochrane Library, and Web of Science from database inception to May 2026. Google Scholar was used as a supplementary source to identify additional relevant articles and citation-linked studies. The search terms included combinations of noble gas-related terms, neuroprotection-related terms, and acquired brain injury-related terms, including “noble gas,” “inert gas,” “xenon,” “argon,” “helium,” “neuroprotection,” “brain protection,” “neuronal injury,” “acquired brain injury,” “traumatic brain injury,” “stroke,” “ischemic stroke,” “middle cerebral artery occlusion,” “cardiac arrest,” “cardiopulmonary resuscitation,” and “hypoxic-ischemic brain injury.”

A total of 254 records were initially identified. After duplicate removal and title abstract screening, full-text articles were reviewed when appropriate. Studies were excluded if they were unrelated to noble gases, did not focus on neuroprotection or acquired brain injury, involved non-neurological injury models, were conference abstracts without sufficient accessible information, were not published in English, or did not provide relevant mechanistic, preclinical, or clinical data. Additional studies were identified by manually checking the reference lists of relevant articles and reviews. Finally, 89 articles were included in this narrative review.

This study focused primarily on preclinical studies and early clinical reports addressing xenon, argon, and helium in acquired brain injury, particularly ischemic stroke, traumatic brain injury, and post-cardiac-arrest hypoxic-ischemic brain injury. Because this was a narrative review rather than a systematic review or meta-analysis, the aim was not to exhaustively include all available studies or perform quantitative pooling, but to provide a focused synthesis of representative evidence. Particular attention was given to studies with clear experimental design, direct comparisons between gases, clinically relevant outcomes, negative or condition-dependent findings, and early translational or clinical evidence.

To ensure a transparent and reproducible selection process, predefined inclusion criteria were applied during full-text screening. Studies were included if they (1) investigated xenon, argon, or helium in the context of acquired brain injury, specifically ischemic stroke, traumatic brain injury, or post-cardiac-arrest hypoxic-ischemic brain injury; (2) employed *in vivo* animal models, *ex vivo* tissue preparations, or early-phase clinical studies; (3) reported at least one relevant outcome, including neurological function, histopathological measures, infarct or lesion volume, neuroinflammatory or apoptotic markers, or safety/feasibility data; and (4) were published as full-length original articles with sufficient methodological detail. Exclusion criteria comprised studies focusing solely on other noble gases (e.g., krypton, neon) without direct translational relevance to the three selected gases, studies limited to non-neurological injury models, conference abstracts without accessible full-text data, and publications in languages other than English. No formal quality assessment or risk-of-bias scoring was performed, as this is a narrative review designed to synthesize representative evidence rather than to provide quantitative effect estimates or meta-analytic pooling. Instead, particular attention was given to studies with clearly defined experimental designs, direct gas comparisons, clinically relevant endpoints, and both positive and negative findings. Studies reporting negative or condition-dependent results were actively sought and included to present a balanced overview and to highlight key sources of heterogeneity, such as variations in injury model, gas concentration, treatment timing, and outcome measures. This approach allows readers to appreciate the context-dependent nature of the available evidence and the substantial gaps that remain before clinical translation can be confidently pursued. Study selection was performed through sequential title/abstract screening followed by full-text evaluation. Discrepancies were resolved through discussion among the authors.

## Xenon

Xenon was a colorless, odorless, tasteless, monatomic noble gas with a relative atomic mass of 131.3. It was present in the atmosphere only in trace amounts, which contributed to its high production cost and limited large-scale clinical use. Nevertheless, xenon had attracted considerable interest in anesthesia, imaging, and neuroprotection because of its distinctive physicochemical and pharmacological properties (Maze and Laitio, 2020).

### Proposed mechanisms of neuroprotection

**FIGURE 1:**
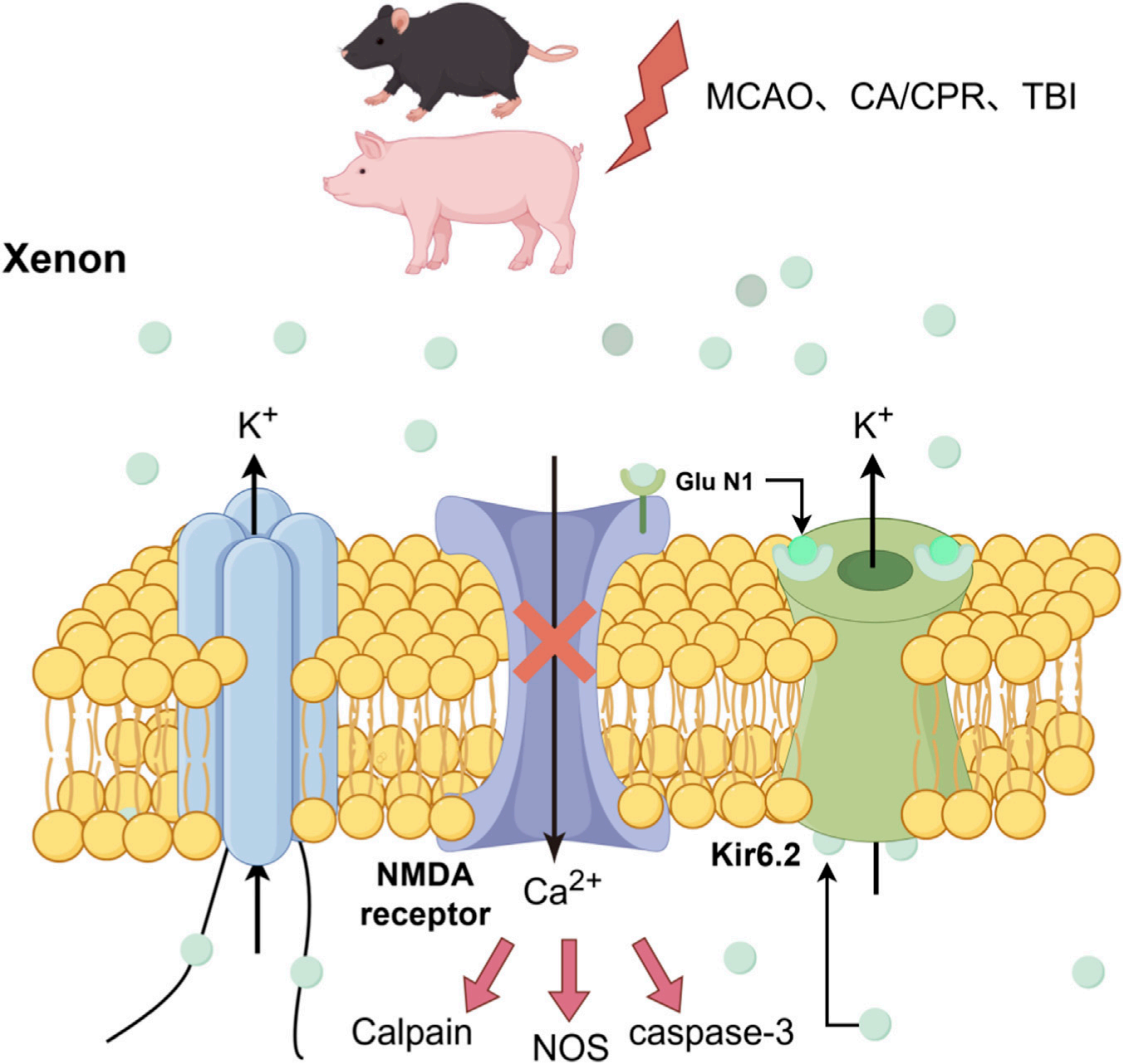
Proposed receptor- and ion-channel-related mechanisms of xenon-mediated neuroprotection. Xenon has been reported to exert neuroprotective effects primarily through inhibition of excitotoxic signaling pathways. Experimental studies demonstrated that xenon acts as a non-competitive antagonist at the NMDA receptor glycine-binding site on the GluN1 subunit, thereby reducing Ca^2+^ influx and downstream activation of neurotoxic cascades including nitric oxide synthase (NOS), calpain, and caspase-3 signaling (Kotani et al., 2023; Harris et al., 2013; Franks et al., 1998). This mechanism was first demonstrated in hippocampal neurons by Franks et al. (1998). Additional studies further suggested that xenon may activate two-pore domain potassium channels (TREK-1) and modulate KATP channels via Kir6.2-containing complexes, leading to reduced glutamate release and neuronal excitability (Gruss et al., 2004; Bantel et al., 2010). In organotypic hippocampal slice models, xenon-mediated protection was reversed by increasing extracellular glycine concentration, further supporting NMDA receptor glycine-site involvement (Banks et al., 2010). These mechanisms are primarily derived from *in vitro* and *in vivo* experimental models and should be interpreted as simplified representations rather than complete signaling pathways. Abbreviations: NMDA, N-methyl-D-aspartate; NOS, nitric oxide synthase; TREK-1, TWIK-related potassium channel 1; KATP, ATP-sensitive potassium channel.

Its neuroprotective effects were thought to be related to pathways involved in ischemic and hypoxic injury after brain damage (Lipton et al., 1994). NMDA receptor activation contributed to neuronal injury through pathways such as NMDA receptor-Ca^2+^ -nitric oxide synthase (NOS) signaling. Xenon-mediated neuroprotection was thought to involve inhibition of the glycine-binding site on the GluN1 subunit of the NMDA receptor (Kotani et al., 2023; Harris et al., 2013). This mechanism was demonstrated in hippocampal neurons by Franks et al. (1998). Another proposed neuroprotective mechanism of xenon involved regulation of TREK-1. TREK-1 was a two-pore-domain potassium channel. Experimental studies had suggested that xenon activates TREK-1 and inhibits voltage-dependent Ca^2+^ channels on the presynaptic membrane, thereby reducing glutamate release after stroke and enhancing NMDA receptor blockade (Gruss et al., 2004). In 2010, Bantel et al. reported that xenon acted as a KATP channel opener by activating Kir6.2, but not Kir6.1 (Bantel et al., 2010). In a mouse organotypic hippocampal slice model of traumatic injury, xenon-mediated neuroprotection was reversed by increasing glycine concentration, supporting competitive inhibition at the NMDA receptor glycine site as an important mechanism (Banks et al., 2010). Figure 1 summarizes the proposed receptor- and ion-channel-related mechanisms of xenon-mediated neuroprotection. Xenon may reduce excitotoxic neuronal injury mainly by inhibiting NMDA receptor-mediated Ca^2+^ influx, particularly through modulation of the glycine-binding site on the GluN1 subunit (Banks et al., 2010). Reduced Ca^2+^ influx may subsequently attenuate downstream activation of NOS, calpain, and caspase-3, thereby limiting neuronal injury. In addition, xenon may activate potassium-channel-related protective pathways, including TREK-1 and Kir6.2/KATP signaling. Compared with argon and helium, xenon currently has the most clearly characterized molecular basis, although its protective effects still depend on concentration, timing, and injury model.

### Experimental and early clinical evidence in acquired brain injury

Because xenon can readily cross the blood-brain barrier, it has been investigated as a potential neuroprotective agent in experimental stroke models. In a mouse transient MCAO model, 70% xenon with 30% oxygen improved functional neurological outcome and reduced total cerebral infarct volume at 24 h compared with nitrous oxide control. In rat temporary focal cerebral ischemia, xenon improved infarct size and neurological scores at 7 days, whereas xenon alone did not improve 28-day outcome. Sustained recovery benefit was observed when xenon was combined with subtherapeutic hypothermia, suggesting that xenon-mediated protection may depend on temperature management and outcome duration (Homi et al., 2003; Sheng et al., 2012). In early rat middle cerebral artery occlusion (MCAO) studies, xenon was reported to improve neurological outcome and reduce infarct volume in the cortex and striatum, particularly at concentrations around 50% (David et al., 2003). Subsequent work in rat transient MCAO models suggested that post-ischemic administration of 50% xenon after 2 h of MCAO also improved outcome (David et al., 2008). In a rat thromboembolic stroke model, xenon was also reported to modulate the effects of recombinant tissue plasminogen activator (rt-PA), reducing rt-PA-associated blood-brain barrier disruption while raising important considerations regarding interaction with thrombolytic therapy (David et al., 2010). In addition, xenon preconditioning improved both histological and neurological outcomes in a transient MCAO model, with benefit observed in both male and female mice (Limatola et al., 2010). Benefit was also reported in a global ischemia model based on bilateral common carotid artery occlusion (BCCAO), in which 50% xenon administered after ischemia reduced neuronal injury and cortical and hippocampal volume loss (Metaxa et al., 2014). More recently, a mouse middle cerebral artery embolism model (eMCAO) was used to evaluate xenon-loaded liposomes as a delivery strategy. In that study, Xe-liposomes administered 2 h after stroke onset, followed by rt-PA at 2 or 4 h, reduced neuronal cell death and appeared to lessen hemorrhagic complications associated with delayed rt-PA treatment (Peng et al., 2023). Taken together, these studies suggested that xenon showed relatively consistent neuroprotective effects in experimental stroke models, particularly when administered early and at concentrations around 50%. However, the magnitude and durability of benefit appeared to depend on the ischemia model, treatment paradigm, and outcome measure, and interactions with thrombolytic therapy require careful interpretation.

Post-cardiac arrest brain injury was another major setting in which xenon had been investigated, because cardiac arrest leads to global cerebral ischemia and hypoxic neuronal injury. Available evidence came mainly from porcine models of cardiac arrest, ventricular fibrillation, and cardiopulmonary resuscitation. In an early porcine study, xenon administered after successful CPR produced only marginal functional improvement, and benefit appeared limited even when treatment was started earlier (Derwall et al., 2008). By contrast, another porcine cardiac arrest study reported that xenon improved histopathological outcome as well as early neurocognitive and neurological function after resuscitation (Fries et al., 2008). In a subsequent porcine study, however, early administration of xenon did not improve neurological or histopathological outcome, highlighting the inconsistency of findings across experimental protocols (Fries et al., 2009). A later porcine study suggested that the combination of xenon with mild therapeutic hypothermia reduced selected histopathological and inflammatory changes more effectively than hypothermia alone, supporting a possible additive effect under these conditions (Fries et al., 2012). Early clinical evidence remained limited. In one randomized study, inhaled xenon combined with hypothermia was associated with reduced white matter injury on MRI, but no significant differences were observed in neurological outcome or mortality at 6 months (Laitio et al., 2016). These findings suggest that imaging benefit does not necessarily translate into established long-term clinical benefit. Overall, preclinical studies suggest that xenon may provide neuroprotection after cardiac arrest, particularly when combined with hypothermia, but the results are not uniformly positive across models. Clinical evidence remains sparse and currently supports, at most, a preliminary imaging-based signal rather than a confirmed improvement in long-term functional recovery or survival (Laitio et al., 2016). Xenon had also been investigated for a potential neuroprotective effect in traumatic brain injury (TBI). In 2013, *in vitro* experiments using organotypic hippocampal slices demonstrated a neuroprotective effect of xenon, whereas argon did not show the same effect, and the underlying mechanism was further explored. In 2015, Campos-Pires et al. used a mouse controlled cortical impact (CCI) model of TBI. In this model, a craniotomy was performed to expose the dura mater over the right parietal cortex, and a pneumatic CCI device was used to produce a standardized moderate injury. Treatment was initiated 15 min, 1 h, 3 h, or 6 h after injury, and animals were randomly assigned to receive 30%, 50%, or 75% xenon, whereas the control group received nitrogen and oxygen. In parallel, mouse organotypic hippocampal slice cultures with focal mechanical injury were used, and cell damage was quantified by propidium iodide fluorescence. The results showed improved neurological outcome and reduced lesion volume after xenon treatment (Campos-Pires et al., 2015). The authors also suggested that pre-injury administration of xenon, as might be relevant in neurosurgical settings, could reduce damage to adjacent healthy tissue. In 2019 and 2020, Campos-Pires et al., using the same CCI model, reported that treatment with 50% or 75% xenon improved neurological outcome and reduced neuronal loss (Campos-Pires et al., 2019; Campos-Pires et al., 2020). In 2018, the same team established an *in vitro* blast-induced injury model using C57BL/6N mouse organotypic hippocampal slice cultures exposed to a single shock wave, and the results were broadly consistent with their earlier findings (Campos-Pires et al., 2018). In 2021, Filiev et al. used a rat weight-drop trauma model in which a 50 g weight was dropped after skull opening, followed by inhalation of 75% xenon for 1 h beginning 15–30 min after injury. In that study, xenon improved motor function and was associated with reduced expression of the inflammatory genes Irf1 and Tlr2 at the injury site 6 h after injury (Filev et al., 2021).

Overall, xenon improved histological or functional outcomes in several rodent and porcine models of acquired brain injury, including cerebral ischemia, post-cardiac-arrest brain injury, and TBI (Table 1). However, these findings were derived predominantly from animal and experimental models. Early clinical studies have emerged in comatose survivors of out-of-hospital cardiac arrest, but current evidence remains preliminary.

**TABLE 1:** Representative xenon studies in acquired brain injury.

No.	Study	Species	Injury model	Intervention	Results	Conclusion
1	2003, H. N. David	Rats	MCAO	50% xenon	Reduced infarct volume in the cortex and striatum	Positive
2	2003, H. M. Homi	Mice	MCAO	70% xenon/30% oxygen	Improved functional and histological outcomes	Positive
3	2008, H. N. David	Rats	MCAO	Post-ischemic xenon after 2 h MCAO	Reduced cortical infarction and improved behavioral outcomes	Positive
4	2004, H. M. Homi	Pigs	7 min of VF and ventilation stopping, 3 min of CPR	Delayed xenon after CPR	Conferred functional neurological improvement despite a 1 h treatment delay	Positive
5	2009, H. N. David	Pigs	8 min of VF and ventilation stopping, 6 min of CPR	Xenon after CPR	Improved histological outcomes and neurocognitive/neurological function	Positive
6	2005, H. M. Homi	Pigs	8 min of VF and ventilation stopping; 6 min of CPR	Early xenon or isoflurane	Did not improve neurological outcomes or cerebral alterations	Negative
7	2010, H. N. David	Rats	MCAO	Xenon in relation to rt-PA thrombolysis	Intra-ischemic xenon inhibited rt-PA-induced thrombolysis, whereas post-ischemic xenon reduced ischemic damage, hemorrhage, and BBB disruption	Conditional
8	2006, H. M. Homi	Mice	tMCAO	Xenon preconditioning	Improved histological and neurological functional outcomes in both sexes	Positive
9	2011, H. N. David	Pigs	10 min of VF and ventilation stopping; 6 min of CPR	Xenon + mild therapeutic hypothermia	Improved histopathological and functional neurological outcomes	Positive
11	2007, H. M. Homi	Rats	Temporary focal ischemia	Xenon ± subtherapeutic hypothermia	Reduced infarct size and improved neurological outcomes; durable benefit was more evident when combined with hypothermia	Conditional
12	2012, H. N. David	Adult OHCA survivors	Xenon + mild therapeutic hypothermia	Xenon + mild therapeutic hypothermia	Xenon administration was feasible and did not cause unexpected serious adverse reactions or major rhythm/conduction abnormalities	Safety
13	2008, H. M. Homi	Rats	MCAO; xenon-containing echogenic liposomes	Xenon-containing echogenic liposomes	Reduced infarct size within a therapeutic window up to 5 h, with an optimal dose range of 7–14 mg/kg	Delivery-based positive
14	2013, H. N. David	Mice	Focal mechanical trauma	Xenon versus argon	Xenon, but not argon, produced neuroprotection; the effect was reversed by increased glycine, supporting involvement of the NMDA receptor glycine site	Direct comparison
15	2009, H. M. Homi	Rats	BCCAO	Delayed post-ischemic xenon	Reduced ischemic neurons and volume loss in the cortex and hippocampus	Positive
16	2014, H. N. David	Comatose survivors after OHCA	Xenon + therapeutic hypothermia	Xenon + therapeutic hypothermia	Reduced MRI-based white matter injury, but 6-month neurological outcome and mortality were not significantly improved	Preliminary clinical signal
17	2015–2020, R. Campos-Pires	Rats	Mice/TBI; controlled cortical impact	30%–75% xenon	Improved neurological outcomes, reduced lesion volume or neuronal loss, and attenuated secondary injury across several TBI experiments	Positive TBI
18	2021, A. D. Filev	Rats	TBI: dosed contusion injury	75% xenon after injury	Improved motor function and was associated with reduced expression of inflammatory genes at the injury site	Positive
19	2021, A. Saraste	110 randomized comatose survivors	Survivors after out-of-hospital cardiac arrest with a shockable rhythm	Xenon + hypothermia	Associated with greater recovery of left ventricular systolic function compared with hypothermia alone	Positive
20	2022, M. P. Dandekar	Rats	MCAO	Repetitive xenon-liposome treatment	Supported safety, behavioral recovery, and reversal of post-stroke brain injury in an extended ischemic model	Positive
21	2023, T. Peng	Rats	Mice/embolic MCAO	Xenon-loaded liposomes + rt-PA	Reduced neuronal cell death and improved neurological recovery	Delivery-based positive
22	2023, S. S. Shin	Pigs	TBI; brain-targeted xenon microbubbles	Brain-targeted xenon microbubbles	Reduced perivascular inflammation and BBB disruption; endothelial experiments suggested preservation of ZO-1	Delivery-based translational positive

Abbreviations: BBB, blood–brain barrier; BCCAO, bilateral common carotid artery occlusion; CCI, controlled cortical impact; CPR, cardiopulmonary resuscitation; MCAO, middle cerebral artery occlusion; MRI, magnetic resonance imaging; OHCA, out-of-hospital cardiac arrest; rt-PA, recombinant tissue plasminogen activator; TBI, traumatic brain injury; tMCAO, transient middle cerebral artery occlusion; VF, ventricular fibrillation; ZO-1, zonula occludens-1.

## Safety considerations: intracranial pressure and clinical boundaries

A critical safety consideration that complicates the clinical translation of xenon is its potential to increase intracranial pressure (ICP). This concern is particularly relevant in acquired brain injury, where elevated ICP and mass effect are major drivers of secondary injury following severe TBI and large hemispheric stroke. The available evidence on xenon and ICP is, however, conflicting and appears to depend heavily on baseline ICP status, xenon concentration, and concomitant sedative or anesthetic agents.

In neurosurgical patients without pre-existing intracranial hypertension, xenon anesthesia has generally been reported as safe. Rylova et al. observed that xenon anesthesia caused only a mild increase in ICP and a modest decrease in cerebral perfusion pressure (CPP), with preserved cerebrovascular reactivity. Similarly, in comatose patients undergoing xenon-CT cerebral blood flow measurement, short-duration inhalation of 28% xenon for 4.5 min did not produce clinically significant ICP changes. A primate study also reported that 33% xenon inhalation did not increase ICP in a cryogenic brain injury model. In contrast, in patients with severe TBI, xenon inhalation (33%, 20 min) for CBF measurement resulted in a clinically meaningful ICP elevation, with a mean increase of 6.9 mmHg and a range of 2–17 mmHg. More direct evidence of dose-dependent risk came from Rylova et al., who studied patients undergoing supratentorial tumor resection. When xenon concentrations reached 55%–65%, 30% of patients—five of whom already had pre-existing intracranial hypertension—developed an abrupt ICP surge to 25 mmHg, leading to premature study termination. This finding establishes a clear safety boundary: in patients with established intracranial hypertension, high-concentration xenon (>50%) may precipitate acute, dangerous ICP elevation and should therefore be avoided.

The underlying mechanisms of xenon-induced ICP elevation are not fully elucidated but may involve a combination of cerebral vasodilation, increased cerebral blood volume, and altered cerebrospinal fluid dynamics. However, it must be emphasized that the neuroprotective evidence for xenon derives primarily from experimental models in which ICP was not a major confounder, such as focal ischemia, cardiac arrest, and controlled cortical impact models without substantial mass effect. Direct clinical evidence that xenon confers net benefit in patients with elevated ICP remains lacking.

Several strategies have been proposed to mitigate ICP risk while preserving potential neuroprotective benefits. First, concentration limitation appears critical, as ICP elevation is dose-dependent; concentrations below 50% appear better tolerated in most patients. Second, combination with ICP-lowering agents, such as propofol, may offset xenon’s pressor effects; Rylova et al. suggested that low-dose xenon combined with low-dose propofol could be a safer alternative. Third, hyperventilation to maintain low CO_2_ levels during xenon administration may help keep ICP and CPP within safer ranges in brain-injured patients. Fourth, invasive ICP monitoring is mandatory in any patient with suspected or established intracranial hypertension who receives xenon, with a predefined discontinuation protocol in the event of acute ICP elevation.

Taken together, the risk-benefit profile of xenon in neuroprotection is not uniform but depends critically on patient selection, baseline ICP status, and treatment protocol. In patients without significant mass effect or intracranial hypertension—such as those with post-cardiac-arrest brain injury—the available evidence suggests that the neuroprotective potential of xenon may outweigh its ICP-related risks when administered at appropriate concentrations and under close monitoring. By contrast, in severe TBI or large hemispheric stroke with established mass effect and elevated ICP, the risk of xenon-induced ICP elevation currently outweighs its unproven neuroprotective benefit, and xenon should be considered contraindicated until further safety data become available.

## Argon

Argon was a noble gas with an atomic number of 18. Argon was a colorless, tasteless, and chemically inert gas, and it was the most abundant noble gas in the atmosphere, with a concentration of approximately 9,340 ppm (B and allentine, 2007).

### Proposed mechanisms of neuroprotection

**FIGURE 2:**
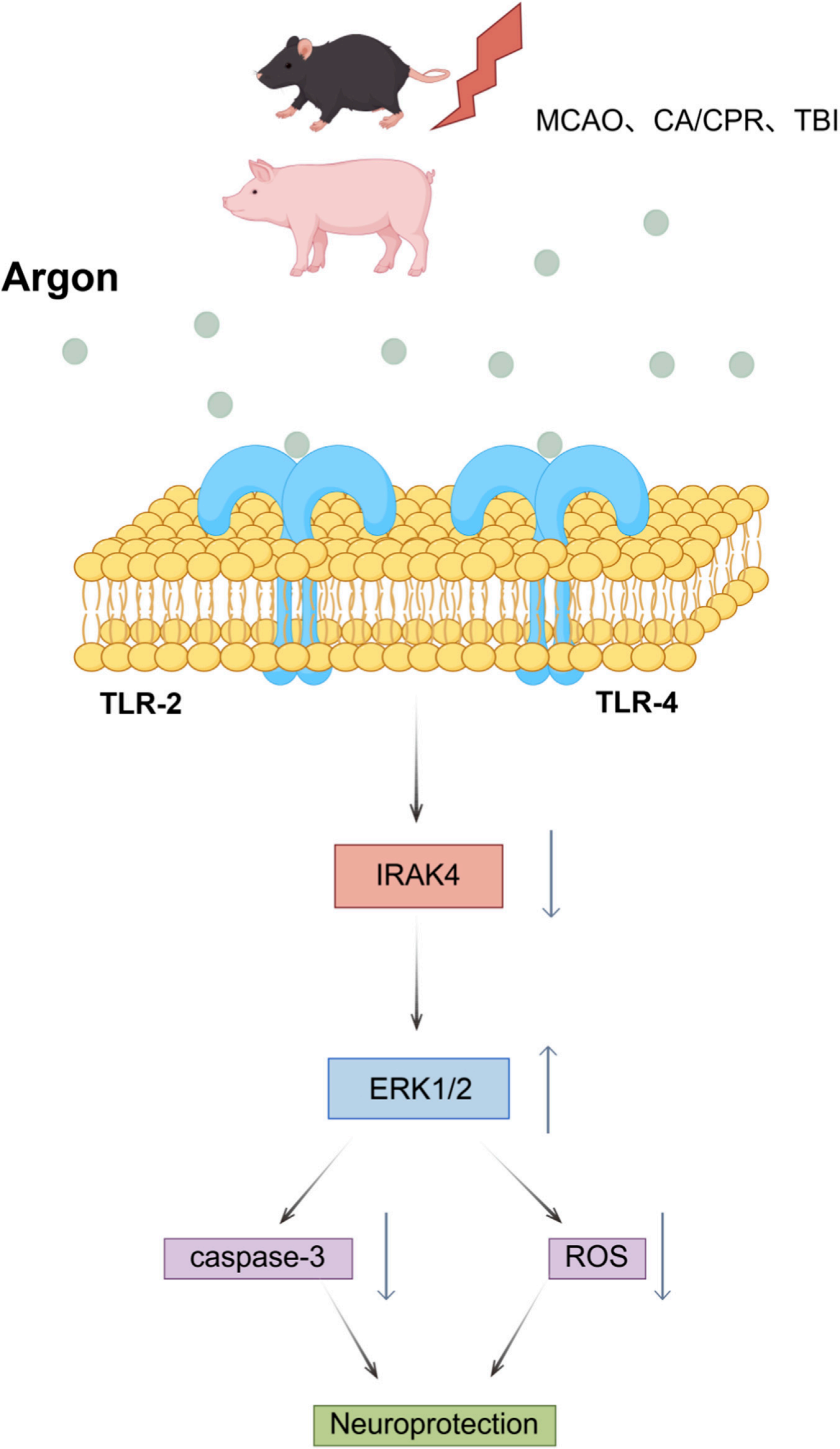
Proposed inflammatory, oxidative, and apoptotic signaling pathways involved in argon-mediated neuroprotection. Argon has been shown to modulate cell survival pathways primarily through regulation of inflammatory and oxidative stress responses. Preclinical studies demonstrated that argon affects Toll-like receptor 2/4 (TLR2/TLR4)-associated signaling, attenuates IRAK4 phosphorylation, and enhances ERK1/2 activation, leading to reduced caspase-3 cleavage and apoptosis (Zhao et al., 2016). Additional studies reported that argon reduces reactive oxygen species (ROS) production and neuronal injury following oxygen-glucose deprivation (Ryang et al., 2011). *In vivo* stroke models further support argon-mediated modulation of inflammatory and injury-related pathways (Ma et al., 2019; Liu et al., 2019; David et al., 2012). However, in cardiac arrest and hypothermia-combination studies, argon showed heterogeneous or even adverse interactions depending on treatment duration and temperature management (Brücken et al., 2017; Brücken et al., 2015). Therefore, the depicted pathways represent a proposed signaling framework based on preclinical evidence, and the direct molecular target of argon remains incompletely defined. Abbreviations: TLR, Toll-like receptor; IRAK4, interleukin-1 receptor-associated kinase 4; ERK1/2, extracellular signal-regulated kinase 1/2; ROS, reactive oxygen species.

The neuroprotective effects of argon were thought to involve reduced apoptosis and attenuation of inflammatory responses. In 2015, Ulbrich et al. reported that argon affected both TLR2 and TLR4 signaling, attenuated IRAK4 phosphorylation, and subsequently increased ERK1/2 phosphorylation. These changes were associated with reduced cleavage and activity of caspase-3, thereby contributing to a cytoprotective effect (Ulbrich et al., 2015). These findings suggested that argon might influence programmed cell death through a series of signaling pathways that converge on caspase-3, thereby reducing apoptosis after acquired brain injury (Figure 2). In 2016, Zhao et al. further showed that reactive oxygen species (ROS) production and caspase-3 activation were reduced in argon-treated neurons exposed to oxygen-glucose deprivation. Additional *in vivo* evidence suggests that argon may exert protective effects in neonatal hypoxic-ischemic injury through activation of Nrf2 signaling. Like xenon, argon can cross the blood-brain barrier, which may facilitate its biological effects in the brain (Zhao et al., 2016). Figure 2 illustrates a proposed inflammatory, oxidative, and apoptotic signaling model for argon-mediated neuroprotection. Argon may modulate TLR2/TLR4-associated signaling, reduce IRAK4 phosphorylation, enhance ERK1/2 phosphorylation, decrease ROS production, and suppress caspase-3 activation. These changes may collectively contribute to reduced secondary neuronal injury. However, unlike xenon, the direct molecular target of argon remains incompletely defined. Therefore, Figure 2 should be interpreted as a proposed signaling framework rather than a definitive receptor-based mechanism.

### Experimental and early clinical evidence in acquired brain injury

In 2011, Ryan et al. reported one of the first *in vivo* stroke models of argon neuroprotection. In that study, rats underwent transient middle cerebral artery occlusion for 2 h and were treated with 50% argon and 50% oxygen. Twenty-four hours later, the argon-treated group showed reduced cortical and basal ganglia infarct volume (Ryang et al., 2011). However, because the observation period in the study by Ryan et al. was relatively short, the durability of argon’s neuroprotective effect could not be fully assessed. In 2019, Ma et al. used a rat permanent focal cerebral ischemia model and extended follow-up to 7 days, again reporting a neuroprotective effect of argon (Ma et al., 2019). In the same year, Liu et al. treated rats with 50% argon for 3 h after transient middle cerebral artery occlusion and 1 h after reperfusion, with neurological assessment performed from 24 h to 7 days (Liu et al., 2019). The results were broadly consistent with those of the previous study, showing reduced neurological deficit during the first week and improved neuronal preservation in the ischemic boundary zone (Liu et al., 2019). However, David et al. reported in 2012 that, after 2 h of MCAO followed by 1 h of 50% argon treatment, cortical infarct volume was reduced whereas subcortical injury increased, and neurological outcome did not improve (David et al., 2012). The authors attributed this discrepancy to methodological factors, including animal movement in the anesthesia chamber and the oxygen concentration used. In a rat tMCAO model, Fahlenkamp et al. reported altered expression of several inflammatory markers and growth factors after tMCAO plus argon compared with tMCAO plus placebo, suggesting that argon may also influence post-ischemic inflammatory and repair-related pathways (Fahlenkamp et al., 2014). In adult ICR mouse tMCAO models, the efficacy of inhaled argon depended strongly on timing, duration, concentration, and ischemia severity, with timely administration during ischemia and shorter exposure durations showing greater benefit than prolonged 24 h treatment. More recently, argon was evaluated in adult *Macaca mulatta* subjected to transient endovascular ischemic stroke, where oxygen-argon treatment reduced ischemic core volume and produced a long-lasting reduction in infarct volume at 1 month, although neurological improvement did not reach statistical significance (He et al., 2022; Torrecilla et al., 2025). Overall, the available evidence for argon in rodent and non-human primate experimental stroke models is mixed (David et al., 2012). These findings suggested that the effect of argon might depend on the ischemia model, treatment duration, and outcome measure.

Argon had also been investigated in models of cardiac arrest and cardiopulmonary resuscitation. In 2013, Brücken et al. used argon in rat models of cardiac arrest, ventricular fibrillation, and cardiopulmonary resuscitation, and reported that 1 h of argon ventilation after successful CPR significantly reduced neuronal injury in the neocortex and hippocampal CA3/4 region (Brücken et al., 2013). In 2014, a comparison of 70% and 40% argon suggested a stronger protective effect at the higher concentration (Brücken et al., 2014). These rat studies suggested that 70% argon may provide neuroprotection after resuscitation from cardiac arrest, although the evidence remains preclinical. In 2014, Ristagno et al. extended this work to a porcine model of cardiac arrest and cardiopulmonary resuscitation (Ristagno et al., 2014). Although the functional results were broadly consistent with those reported by Brücken et al., pathological benefit was less clear in the porcine model (Ristagno et al., 2014). The authors suggested that this discrepancy might be related to the timing of assessment and the brain regions selected for histopathological evaluation, noting that the putamen and caudate nucleus might be more sensitive indicators of injury than the hippocampal CA1 region in this setting (Ristagno et al., 2014).

In 2016, Zuercher et al. evaluated 50% argon for 24 h in a rat cardiac arrest model and found no improvement in outcome compared with controls (Zuercher et al., 2016). The authors suggested that the severity of injury and the timing or duration of argon administration might have contributed to this negative result (Zuercher et al., 2016). These findings suggested that prolonged argon administration might not be beneficial under all conditions and could even be detrimental in some settings. In 2017, Brücken et al. studied argon in combination with mild therapeutic hypothermia (MTH) in a rat cardiac arrest model and found that this combination did not improve neurological outcome and may even have worsened neurological injury (Brücken et al., 2017). The authors proposed that argon inhalation after MTH might interfere with temperature modulation, which could partly explain the unfavorable neurological outcome. However, the relationship between argon and temperature regulation remained uncertain and requires further investigation. Notably, this negative interaction with hypothermia stands in marked contrast to the consistently additive or synergistic effects observed when xenon is combined with MTH in similar experimental settings, suggesting that the biological interaction of different noble gases with temperature management is gas-specific and may involve fundamentally distinct mechanisms. Delayed treatment also appears relevant. In male Sprague-Dawley rats subjected to cardiac arrest and CPR, 1 h of 70% argon ventilation improved neurological deficit scores and reduced neuronal damage even when administration was delayed for 3 h after return of spontaneous circulation. In a severe porcine cardiac arrest model with 12 min of untreated arrest, 4 h of post-resuscitation argon ventilation improved 96-h survival and good neurological recovery, with greater benefit observed at 70% argon than at 50% argon (Brücken et al., 2015; Fumagalli et al., 2020). Taken together, the evidence for argon after cardiac arrest was heterogeneous. Some animal studies suggested neuroprotective effects, whereas others report neutral or even unfavorable outcomes depending on treatment duration, injury severity, and interaction with hypothermia (Zuercher et al., 2016; Brücken et al., 2017).

Argon has also been investigated in experimental TBI models, but the findings are inconsistent. In mouse organotypic hippocampal slice models of TBI or oxygen-glucose deprivation, 25%, 50%, and 74% argon reduced cell damage, and argon treatment initiated within 2 h after trauma attenuated secondary injury in mouse pup organotypic hippocampal slice models (Loetscher et al., 2009; Grüßer et al., 2017). *In vivo* findings were more variable. In a mouse CCI model, early administration of 70% argon for 24 h was associated with reduced edema, improved sensorimotor recovery, better memory performance, and a trend toward increased white matter integrity (Moro et al., 2021). However, in male Wistar rats with open TBI, 3 h inhalation of 70% argon/30% oxygen did not improve neurological tests, MRI-based lesion volume, or histological tissue loss at 14 days (Antonova et al., 2022). Similarly, in adult male mice with closed-head injury, 24 h inhalation of 70% or 79% argon did not improve neurological recovery, body-weight recovery, neurodegeneration, or neuroinflammation up to 36 days (Creed et al., 2021). In another mouse CCI study, 4 h of argon treatment did not reduce contusion volume or edema, although it reduced intracranial pressure and improved selected beam-walk measures (Schneider et al., 2022). These findings suggest that argon-mediated effects in TBI are highly dependent on injury model, exposure duration, gas concentration, and selected outcome measures (Antonova et al., 2022; Creed et al., 2021). Taken together, available studies suggest that argon may exert neuroprotective effects in some TBI models, particularly when administered early, although the results remain inconsistent across experimental settings.

Overall, as summarized in Table 2, neurological scoring tended to favor argon-treated animals in several studies, although statistically significant functional benefit was not consistently demonstrated. Moreover, one preclinical study (Barros et al., 2025) reported that both xenon and argon showed neuroprotective effects in models of perinatal hypoxic-ischemic encephalopathy. However, perinatal hypoxic-ischemic encephalopathy lies outside the main scope of the present review. In the clinical setting, a protocol for the CardioPulmonary resuscitation with Argon (CPAr) trial has been published, indicating that clinical evaluation of argon during cardiopulmonary resuscitation is being planned or initiated (Ristagno et al., 2025). However, clinical outcome data remain unavailable. Overall, argon remains a promising but less well-established candidate than xenon. Preclinical studies in stroke, cardiac arrest, and TBI models suggest possible neuroprotective effects under selected conditions, but the available evidence remains heterogeneous and sometimes contradictory.

**TABLE 2:** Representative argon studies in acquired brain injury.

No.	Study	Species	Injury model	Intervention	Results	Conclusion
1	2009, Philip D Loetscher	Organotypic hippocampal slice cultures from mice/Pups	OGD and focal mechanical trauma	25%–74% argon	Argon reduced cell injury in *in vitro* models of cerebral ischemia and traumatic injury	*In vitro* positive
2	2011, Ryang	Rats	MCAO	50% argon/50% oxygen	Reduced infarct volume and composite adverse outcomes	Positive stroke
3	2012, David	Rats	MCAO	50% argon	Reduced cortical injury but increased subcortical injury; neurological outcome did not improve	Conditional
4	2013, A. Brücken	Rats	VF with ventilation arrest followed by CPR	Argon after CPR	Improved histopathological and functional neurological outcomes	Positive
5	2014, A. Brücken	Rats	VF with ventilation arrest followed by CPR	40% or 70% argon	Reduced neurological impairment and neuronal damage index, with stronger effects at higher concentration	Positive
7	2014, Ristagno	Pigs	VF with ventilation arrest followed by CPR	Post-resuscitation argon	Improved neurological outcome; histological benefit was less clear than functional recovery	Positive
8	2015, A. Brücken	Rats	VF with ventilation arrest followed by CPR	Delayed 70% argon after ROSC	Improved neurological deficit scores and reduced neuronal damage even when administration was delayed	Positive
9	2016, Zuercher	Rats	Cardiac arrest followed by CPR	50% argon or 50% helium for 24 h	Neither argon nor helium improved functional outcome or reduced neuronal damage in the hippocampal CA1 region	Negative
10	2017, A. Brücken	Rats	Cardiac arrest + MTH	Argon + mild therapeutic hypothermia	Neuronal damage index increased and neurological outcome did not improve	Negative
11	2017, Linda Grüßer	The organotypic hippocampal slices from pups of 5- to 7-day-old mice	*In vitro* TBI model	Argon during early post-traumatic incubation	Shorter exposure periods produced different outcomes, supporting further investigation of the first hours after TBI.	Supportive
12	2019, Liu	Rats	MCAO	Post-stroke argon treatment	Alleviated neurological deficit and neuronal loss	Positive
13	2019, Ma	Rats	tMCAO/pMCAO	Argon inhalation after ischemia	Improved neurological outcome, overall recovery, and infarct volume	Positive
14	2020, Fumagalli	Pigs	Prolonged VF with ventilation arrest followed by CPR	50% or 70% argon	Improved neurological recovery and ameliorated brain injury, with greater benefit after 70% argon	Positive
15	2021, Creed	Mice	TBI/closed-head injury	70% or 79% argon for 24 h	Did not improve neuronal quantification, recovery, neuroinflammation, or neurological outcome	Negative
16	2021, Moro	Mice	TBI/CCI	Early 70% argon	Improved sensorimotor function and cognitive/structural outcomes	Positive
17	2022, J. He	Mice	tMCAO	Inhaled argon with different timing/duration protocols	Timely argon improved lesion volume, edema, and neurological scores; shorter exposure durations were more effective than prolonged 24 h treatment	Conditional
18	2022, V. V. Antonova	Rats	Open TBI	70% argon/30% oxygen for 3 h	Did not improve neurological tests, MRI lesion volume, or histological tissue loss at 14 days	Negative
19	2022, F. I. Schneider	Mice	TBI/CCI	Argon for 4 h	Did not reduce contusion volume or edema, but reduced intracranial pressure and improved selected beam-walk measures	Conditional
20	2025, S. G. Torrecilla	*Macaca mulatta*	Transient endovascular ischemic stroke	Oxygen-argon treatment	Reduced ischemic core volume and produced sustained reduction in infarct volume at 1 month, although neurological improvement was not significant	Translational positive

Abbreviations: MCAO, middle cerebral artery occlusion; tMCAO, temporary middle cerebral artery occlusion; pMCAO, permanent middle cerebral artery occlusion; VF, ventricular fibrillation; CPR, cardiopulmonary resuscitation; TBI, traumatic brain injury; CCI, controlled cortical impact; CHI, closed head injury; BCCAO, bilateral common carotid artery occlusion; eMCAO, endovascular middle cerebral artery occlusion. CA, cardiac arrest; CPR, cardiopulmonary resuscitation.

## Helium

Helium was the first noble gas in the periodic table, and its atmospheric concentration was extremely low, at approximately 0.000524% (Martinón-Torres et al., 2002). Because helium was less dense than oxygen, helium-oxygen mixtures were commonly used in respiratory care, particularly in infants with airway or respiratory distress (Martinón-Torres et al., 2002). The possible role of helium in neuroprotection had also been explored.

### Proposed mechanisms of neuroprotection

**FIGURE 3:**
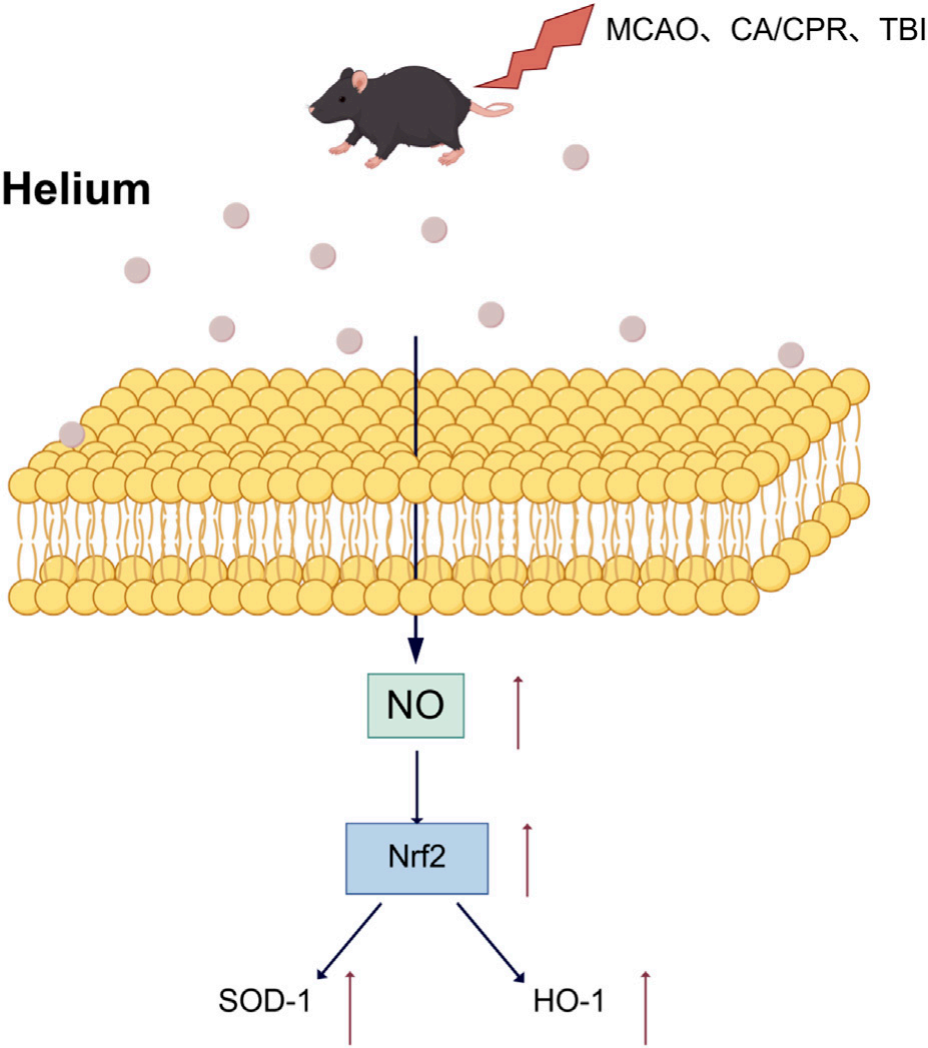
Proposed condition-dependent mechanisms of helium-related neuroprotection. Unlike xenon, helium is not an anesthetic gas and does not have a defined molecular receptor target. Experimental studies suggest that helium preconditioning may attenuate hypoxia-ischemia-induced brain injury via caspase-3 inhibition and activation of nitric oxide (NO)-dependent Nrf2 antioxidant signaling pathways, including upregulation of heme oxygenase-1 and superoxide dismutase-1 (Li et al., 2016b). Additional studies indicate that helium may modulate the neurovascular niche by reducing inflammatory cytokines and enhancing angiogenesis-related factors (David et al., 2009). Importantly, several studies have shown that helium-mediated neuroprotection is highly temperature-dependent, and may largely reflect hypothermia-related effects rather than direct molecular interactions (Aehling et al., 2018). Therefore, helium-related biological effects appear strongly dependent on experimental conditions including temperature, pressure, timing, and concentration, and should be interpreted as a simplified, hypothesis-generating framework rather than a definitive mechanistic model. Abbreviations: NO, nitric oxide; Nrf2, nuclear factor erythroid 2-related factor 2; SOD-1, superoxide dismutase 1; HO-1, heme oxygenase-1.

Unlike xenon, helium was not an anesthetic gas and had mainly been studied in experimental rather than clinical neuroprotection. In clinical practice, helium was more commonly used as part of a helium-oxygen mixture than as a neuroprotective intervention. In neonatal rat hypoxia-ischemia models, helium preconditioning (He-PC) had been reported to reduce infarct volume and the number of apoptotic cells, possibly through inhibition of caspase-3 activity (Liu et al., 2011). In neonatal rat hypoxia-ischemia models, Li et al. further reported that helium pretreatment promoted nitric oxide (NO) production, which activated Nrf2 signaling and increased expression of the downstream antioxidant enzymes heme oxygenase-1 and superoxide dismutase-1 (Li et al., 2016a). Additional work suggested that helium preconditioning may also improve the neurovascular niche by reducing pro-inflammatory cytokines, increasing neurotrophic and angiogenesis-related factors, and promoting microvessel formation (Li et al., 2016b). Another proposed mechanism involved temperature-dependent effects (David et al., 2009). In adult rat MCAO-induced cerebral ischemia, David et al. reported that helium could provide neuroprotection in MCAO-induced cerebral ischemia under selected temperature conditions, apparently through induction of hypothermia. In that study, helium treatment at 25 °C reduced infarct volume and motor deficits, whereas treatment at 33 °C showed no neuroprotective effect (David et al., 2009). These findings suggest that the biological effects of helium depend strongly on experimental conditions rather than on chemical inertness alone (Figure 3). Figure 3 summarizes the proposed mechanisms helium-related neuroprotection. Unlike xenon, whose NMDA receptor-related mechanisms have been relatively well characterized, the direct molecular target of helium remains unclear. Current evidence suggests that helium may exert biological effects under selected experimental conditions through modulation of NO production, Nrf2-related antioxidant signaling, mitochondrial responses, membrane-associated biophysical processes, and temperature-dependent cellular stress responses. Therefore, the helium-related pathway shown in Figure 3 should be regarded as a simplified mechanistic framework rather than a definitive molecular mechanism.

### Experimental evidence in acquired brain injury

The number of neuroprotection studies on helium remained limited, but the available evidence could be grouped into ischemia-related models, cardiac arrest or CPR models, and trauma models. In an early rat MCAO study, 70% helium reduced cerebral infarct volume, whereas 40% helium did not, suggesting a concentration-dependent effect (David et al., 2009). In 2018, Aehling et al. used a rat ventricular fibrillation/resuscitation model to examine helium preconditioning and postconditioning, reporting limited change in neuronal counts but some improvement in apoptosis-related measures (Aehling et al., 2018). In neonatal rat hypoxic-ischemic models, helium had also been associated with increased cortical and hippocampal neuronal survival, improved sensorimotor and cognitive function, and reduced infarct size and caspase-3 activity (Liu et al., 2011). Together, these findings suggested that the apparent benefit of helium depends strongly on treatment timing and duration. By contrast, Zuercher et al. found no clinical or histological neuroprotection after 24 h of 50% helium treatment following 8 min of cardiac arrest (Zuercher et al., 2016). In a mouse organotypic hippocampal slice model of focal mechanical injury, Coburn et al. reported that elevated helium pressure provided neuroprotection under selected pressure and temperature conditions (Coburn et al., 2008). Compared with xenon, the role of helium under different atmospheric or admixture conditions remained less well defined. In 2016, Haelewyn et al. explored helium administered with tissue plasminogen activator (tPA) as a reperfusion-related strategy, suggesting that helium-based combinations might influence the effects of thrombolytic treatment (Haelewyn et al., 2016). These findings are of interest because they imply that helium may not act only through direct cellular protection, but may also affect the broader reperfusion environment after ischemic injury. Although helium might share some biological features with xenon, its neuroprotective evidence base was much smaller and requires further experimental validation. In adult male rat transient focal cerebral ischemia, 70% helium/30% oxygen administered immediately after MCAO reduced infarct size and improved neurological deficit scores, whereas delayed administration or lower helium concentration showed weaker effects. However, in a randomized rat cardiac arrest model, 24 h exposure to 50% helium after resuscitation did not improve histological or neurobehavioral outcomes. In a neonatal rat asphyxia model directly comparing argon, helium, and xenon, helium improved outcomes after moderate injury but showed a weaker protective profile than argon or xenon after severe injury. These findings suggest that helium-related neuroprotection is strongly dependent on timing, concentration, injury severity, and model type (Zuercher et al., 2016; Pan et al., 2011; Brevoord et al., 2016; Zhuang et al., 2012). The Xe-He-37.5 mixture has been proposed as a xenon-sparing strategy because it reduced oxygen-glucose deprivation-induced injury in brain slices while also inhibiting tissue plasminogen activator activity. This finding suggests that Xe-He mixtures may have translational potential, but their timing relative to thrombolytic therapy requires careful evaluation (David et al., 2017). This mixed-gas strategy is potentially attractive because it may preserve some beneficial properties of xenon while reducing the amount of xenon required. However, this approach remained experimental, and its safety, reproducibility, and translational feasibility have not yet been established. Protective effects of Xe-He-37.5 had also been explored in other organs (Table 3). Overall, helium remained much less studied than xenon or argon. Available findings suggested possible neuroprotective effects under selected experimental conditions, but the current evidence remained limited and further research was required.

**TABLE 3:** Representative helium and comparative noble gas studies in acquired brain injury.

No.	Study	Species	Injury model	Intervention	Results	Conclusion
1	2011, Y. Pan	Rats	Focal transient cerebral ischemia/MCAO	Heliox	Reduced infarct volume and improved 24 h neurological deficits	Positive
2	2008, Mark Coburn	Mouse pup organotypic hippocampal brain slices	*In vitro* TBI model	Helium or xenon under selected pressure/temperature conditions	Helium showed neuroprotective effects under selected pressure and temperature conditions; xenon showed stronger or more consistent protection	Conditional *in vitro* positive
3	2009, H. N. David	Rats	Transient MCAO	75% helium	Produced neuroprotection and improved neurological outcome by inducing hypothermia due to high specific heat compared with air	Positive
4	2011, Y. Liu	Neonatal rats	Hypoxia-ischemia	Helium preconditioning	Reduced infarct ratio, increased neuronal survival, inhibited apoptosis and caspase-3 activity, and improved long-term sensorimotor/cognitive outcomes	Positive
5	2012, L. Zhuang	Neonatal rats	Hypoxia-ischemia/neonatal asphyxia	Argon, helium, or xenon	All gases improved outcomes after moderate injury; under severe injury, helium showed a weaker protective profile than argon or xenon	Conditional
6	2016, B. Haelewyn	Rats	Thromboembolic stroke	Helium with tPA-related treatment paradigms	Helium should not be administered before or with tPA because it may inhibit tPA-induced thrombolysis; it may be protective after tPA-induced reperfusion	Conditional
7	2016, P. Zuercher	Rats	Cardiac arrest followed by CPR	50% helium or 50% argon for 24 h	Neither argon nor helium improved functional outcome or reduced neuronal damage in the hippocampal CA1 region	Negative
8	2016, Y. Li	Neonatal rats	Hypoxia-ischemia	Helium preconditioning	Reduced pro-inflammatory cytokines, increased neurotrophic and angiogenesis-related factors, and promoted microvessel formation	Positive
9	2016, Y. Li	Neonatal rats	Hypoxia-ischemia	Helium preconditioning	Promoted NO production, activated Nrf2 signaling, and increased HO-1 and SOD-1 expression	Positive
10	2016, D. Brevoord	Patients after cardiac arrest	Post-cardiac arrest syndrome	3 h ventilation with 1:1 helium/oxygen mixture	Feasible and not associated with helium-related adverse events, but efficacy was not established	Safety
11	2017, H. N. David	Brain slices/*ex vivo* model	OGD model of acute ischemic injury	Xe-He-37.5 mixture	Reduced OGD-induced injury but inhibited rt-PA activity, suggesting that timing relative to thrombolysis requires caution	Conditional
14	2018, C. Aehling	Rats	Resuscitation model	Helium pre-/post-conditioning	Showed histological/apoptosis-related signals associated with Cav-1 and Cav-3, but did not improve functional outcome or viable neuron count	Positive

Abbreviations: Cav, caveolin; CPR, cardiopulmonary resuscitation; HO-1, heme oxygenase-1; MCAO, middle cerebral artery occlusion; NO, nitric oxide; Nrf2, nuclear factor erythroid 2-related factor 2; OGD, oxygen–glucose deprivation; rt-PA, recombinant tissue plasminogen activator; SOD-1, superoxide dismutase 1; TBI, traumatic brain injury; Xe-He-37.5, xenon–helium gas mixture at equimolar concentration of 37.5%.

Taken together, the three mechanistic diagrams represent different levels of mechanistic certainty. Xenon has the strongest mechanistic support, with relatively specific receptor- and ion-channel-related pathways. Argon appears to involve broader inflammatory, oxidative, and apoptotic signaling, but its direct molecular target is still unclear. Helium has the most exploratory mechanism, and its apparent effects seem highly dependent on temperature, pressure, concentration, and timing. Therefore, these diagrams should be interpreted as simplified mechanistic summaries rather than complete or definitive signaling maps.

## Other noble gases

In addition to xenon, argon, and helium, several other noble gases have also been explored in experimental settings (Table 4). Krypton has been reported to reduce focal ischemic brain injury in one experimental study, suggesting that noble gases beyond xenon may also exert biological effects in the injured brain (Antonova et al., 2024). However, the currently available evidence for krypton remains very limited, and the underlying mechanisms have not been adequately characterized. In contrast, neon did not show clear neuroprotective effects in the available cell-based experiments (Koziakova et al., 2019), which suggests that not all noble gases share the same biological potential in neural injury models. This difference also indicates that neuroprotective activity cannot be assumed solely on the basis of chemical inertness. Rather, any biological effect is likely to depend on specific physicochemical properties, experimental conditions, and the injury model used. At present, studies of krypton, neon, and other less-investigated noble gases remain sparse, and most findings are restricted to isolated experimental observations without sufficient replication or translational relevance. Therefore, the current evidence is insufficient to support meaningful comparison of these gases with xenon, argon, or helium, all of which have been investigated more extensively in acquired brain injury models. For this reason, these other noble gases are not discussed further in the present review.

**TABLE 4:** Other noble gases briefly explored in neural injury models.

No.	Study	Species	Injury model	Intervention	Reuslts	Conclusion
1	2019, Mariia Koziakova	Mouse organotypic hippocampal slice cultures/isolated cell cultures	Hypoxia–ischemia, OGD, or traumatic insult	Krypton or neon	Krypton and neon did not show protective effects, unlike xenon and argon in related experimental contexts	Negative
2	2024, Viktoriya V Antonova	Male Wistar rats	Photothrombotic ischemic stroke	Krypton inhalation	Krypton inhalation reduced neurological impairment and ischemic damage in one experimental study; proposed signaling pathways require further validation	Positive

Abbreviations: OGD, oxygen–glucose deprivation.

## Discussion

Noble gases have a wide range of biological properties, including anesthetic, analgesic, anti-apoptotic, cytoprotective, and potentially neuroprotective effects. However, their established clinical use remains primarily anesthetic rather than neuroprotective. In acquired brain injury, xenon, argon, and helium have been explored mainly in stroke, traumatic brain injury (TBI), and post-cardiac-arrest brain injury models, where secondary injury processes such as excitotoxicity, inflammation, oxidative stress, apoptosis, and blood-brain barrier disruption contribute to neurological damage. Overall, the biological effects of noble gases appear to depend on gas type, concentration, timing of administration, treatment duration, and injury model. Among the noble gases reviewed, xenon currently has the strongest and most consistent evidence base. Xenon is an approved anesthetic agent and has been investigated clinically as a possible alternative to nitrous oxide. Preclinical studies support its neuroprotective effects in stroke, post-cardiac-arrest brain injury, and TBI models. In stroke models, xenon has shown potentially beneficial effects, including reduced infarct volume, improved neurological outcome, and possible mitigation of blood-brain barrier disruption and hemorrhagic complications associated with delayed rt-PA treatment. In post-cardiac-arrest models, xenon had also shown benefit in some studies, particularly in combination with mild therapeutic hypothermia (MTH). Nevertheless, the evidence was not completely uniform, and even for xenon, the magnitude and durability of benefit appear to depend on injury model, treatment timing, concentration, and outcome measure. In addition, the effective concentrations used experimentally were often relatively high, commonly around 50% or 70%, which might limit feasibility in patients who require high oxygen concentrations. Thus, a balance between oxygen demand and xenon delivery remained necessary. Moreover, xenon faces important practical barriers, including high cost, limited availability, gas-recycling requirements, and the challenge of maintaining adequate oxygen delivery while using high xenon concentrations. Therefore, xenon should currently be regarded as the most promising noble gas for neuroprotection, but not yet as an established neuroprotective therapy in routine clinical practice (Dingley et al., 2015).

Argon has been studied less extensively than xenon and was not an approved anesthetic agent. Experimental studies suggested that argon might exert neuroprotective effects under selected conditions, possibly through reduced apoptosis and modulation of inflammatory signaling pathways. However, compared with xenon, the evidence for argon remains more heterogeneous and sometimes contradictory. In stroke models, several studies reported reduced infarct volume and improved neurological outcome, whereas others found no functional benefit or even region-specific worsening despite partial histological improvement. Similarly, in cardiac arrest models, some studies suggested neuroprotection after resuscitation, but others reported neutral or even unfavorable outcomes, particularly in relation to treatment duration and interaction with hypothermia. In animal studies of cardiac arrest, argon combined with MTH did not improve outcome and might even have had unfavorable effects, possibly because of interference with temperature regulation, although the mechanism remains uncertain. In 2020, Benedikt Kremer reported that argon might affect the microglial inflammatory response after subarachnoid hemorrhage (Kremer et al., 2020), suggesting that its biological effects might extend beyond ischemic models. In TBI models, argon showed potentially beneficial effects in some *in vitro* and *in vivo* studies, especially when administered early, but negative findings had also been reported after prolonged treatment. Overall, argon remains a promising but less well-established candidate than xenon. Future studies should first clarify the optimal timing, exposure duration, concentration, and interaction with temperature management before large efficacy trials are considered.

A recent preclinical systematic review and meta-analysis by Liang et al. (2022) quantitatively synthesized 32 studies and reported that both argon and xenon exhibited significant positive neuroprotective effect sizes across cardiac arrest, traumatic brain injury, and stroke models, with xenon showing a significantly larger overall effect size (34.1%; 95% CI, 24.7%–43.6%) than argon (18.1%; 95% CI, 8.1%–28.1%). These quantitative findings are broadly consistent with the qualitative assessment of the present narrative review, which also identified xenon as having the strongest and most consistent evidence base, whereas argon’s evidence remains more heterogeneous. However, the meta-analytic approach necessarily pooled data across diverse experimental conditions, whereas the present review emphasizes that the magnitude and even direction of neuroprotection for both gases depend critically on injury model, treatment timing, concentration, exposure duration, and interaction with concomitant therapies such as hypothermia—nuances that a pooled estimate alone cannot fully capture.

The amount of helium research is much smaller than that of xenon and argon. Helium is not an anesthetic gas, and its possible neuroprotective effects have mainly been investigated in experimental settings. Available preclinical evidence suggests that helium-related biological effects are highly condition-dependent. Helium preconditioning has been reported to attenuate hypoxia-ischemia-induced brain injury in neonatal rat models, possibly through caspase-3 inhibition, NO/Nrf2-related antioxidant signaling, and modulation of the neurovascular niche (Liu et al., 2011; Li et al., 2016a; Li et al., 2016b). In adult rat MCAO models, helium-related protection appeared to be temperature- and concentration-dependent, as 70% helium but not 40% helium reduced infarct volume, and the protective effect was linked to hypothermia-producing conditions (David et al., 2009). In addition, helium showed neuroprotection under elevated pressure and temperature-controlled conditions in an *in vitro* traumatic brain injury model 2, whereas prolonged 50% helium exposure failed to improve clinical or histological outcomes in a rodent cardiac arrest model (Zuercher et al., 2016). These findings indicate that helium is unlikely to be a robust stand-alone neuroprotective gas at the current stage. Instead, it may be better considered as a condition-sensitive biological modulator that requires strict control of temperature, pressure, concentration, timing, and exposure duration. The Xe-He-37.5 mixture has been proposed as a xenon-sparing strategy, but its safety, reproducibility, and interaction with reperfusion therapy remain experimental questions (David et al., 2017).

When the three gases are compared more broadly, an important distinction emerges between biological plausibility, consistency of evidence, and translational feasibility. Xenon currently occupies the strongest position because it combines a relatively clear mechanistic basis with the broadest preclinical support and the most developed early clinical evidence, although high cost and oxygen-delivery constraints limit routine use. Argon is more accessible and potentially more practical, but its evidence base is less coherent and its mechanisms remain less clearly defined. Helium is the most exploratory candidate, with possible value mainly in preconditioning, carefully timed adjunctive therapy, mixed-gas strategies, or mechanistic studies rather than immediate stand-alone clinical application.

## The paradox of noble gas–hypothermia interactions: xenon versus argon

A noteworthy and mechanistically informative paradox emerges when comparing the effects of xenon and argon in combination with mild therapeutic hypothermia (MTH). In several preclinical studies, xenon combined with MTH has consistently shown additive or synergistic neuroprotection, particularly in cardiac arrest models, where the combination improved both histopathological and functional outcomes to a greater extent than either intervention alone. This positive interaction has also received preliminary clinical support, with xenon–hypothermia combination showing reduced MRI-based white matter injury in comatose survivors of out-of-hospital cardiac arrest. In contrast, the combination of argon with MTH not only failed to provide additional benefit but may have exacerbated neurological injury in a rat cardiac arrest model.

Several interconnected hypotheses may account for this dichotomy. First, the most plausible explanation lies in the divergent primary molecular targets of the two gases. Xenon acts predominantly as a non-competitive antagonist at the NMDA receptor glycine site, directly attenuating excitotoxic calcium influx—a pathway that is central to secondary injury and is known to be synergistically suppressed by hypothermia through reduced glutamate release and energy metabolism. Argon, by contrast, appears to exert its effects primarily through modulation of TLR2/TLR4-associated inflammatory signaling and oxidative stress responses, rather than directly interfering with excitotoxic glutamatergic transmission. These distinct signaling frameworks may interact with hypothermia-induced cellular stress responses (e.g., heat shock proteins, cold-inducible RNA-binding proteins) in fundamentally different ways, potentially leading to pathway-specific synergy in the case of xenon, and detrimental crosstalk or signal interference in the case of argon. Second, a thermoregulatory interference mechanism cannot be excluded. Brücken et al. raised the possibility that argon may interfere with the induction or maintenance of therapeutic hypothermia, an effect that has not been reported for xenon (Brücken et al., 2015). If argon alters peripheral thermoregulation or central temperature set-point modulation, it could offset the protective effects of targeted temperature management, thereby masking or reversing argon’s intrinsic neuroprotective potential under normothermic conditions. Third, differences in the therapeutic time-window and effective concentration under hypothermic conditions may also contribute. Hypothermia alters cerebral blood flow, blood-brain barrier permeability, and tissue oxygen metabolism, which may shift the effective concentration–response curve of argon, rendering a normally protective concentration ineffective or even deleterious when combined with cooling. These hypotheses are not mutually exclusive and highlight the need for targeted mechanistic studies that directly compare the two gases under strictly controlled temperature protocols.

This paradox carries important translational implications. First, it strongly suggests that neuroprotective efficacy observed under normothermic conditions cannot be extrapolated to hypothermic settings, and *vice versa*. Second, it underscores the necessity of gas-specific and temperature-protocol-specific optimization in future preclinical studies and clinical trial design. Future studies should systematically examine these interactions using standardized temperature protocols, extended outcome measures, and, where possible, direct head-to-head comparisons between xenon, argon, and other noble gases under identical experimental conditions.

## Risk–benefit trade-off in xenon neuroprotection: balancing efficacy against intracranial hypertension

Currently, no established preclinical or early clinical benchmark exists that quantitatively defines a “net benefit” threshold for xenon in the presence of intracranial hypertension. The available data, however, allow for a qualitative risk stratification. In patients with normal baseline ICP and preserved intracranial compliance, the risk appears acceptable, and the neuroprotective evidence—though largely preclinical—suggests a favorable risk-benefit orientation. In patients with known intracranial hypertension or severe mass effect, the risk is substantially elevated, and the lack of direct clinical evidence for net benefit makes the risk-benefit balance distinctly unfavorable. This stratification is supported by dose-dependence; higher xenon concentrations (>50%) are associated with greater ICP elevation, whereas lower concentrations (≈30%) appear better tolerated. Furthermore, the therapeutic window for xenon in most experimental models is narrow (within hours of injury), which may limit the time available for stabilization of ICP before treatment initiation.

It is also important to recognize that the neuroprotective evidence for xenon is predominantly derived from animal models in which ICP was either not measured or not a significant confounder. In models of transient focal ischemia and cardiac arrest, the primary injury mechanisms are excitotoxicity and oxidative stress rather than mass effect, making them poor proxies for the clinical reality of severe TBI or malignant stroke. Therefore, the translational leap from these models to patients with elevated ICP is inherently precarious.

Until more definitive safety data become available, the prudent translational approach is to restrict clinical investigation of xenon to patient populations without significant intracranial hypertension, such as post-cardiac-arrest survivors with preserved compliance. In TBI and stroke patients with mass effect, xenon should be approached with extreme caution, if at all, and only under strict ICP monitoring with predefined safety thresholds and early termination criteria. Future research should prioritize the systematic collection of ICP data in all preclinical and early-phase clinical studies to enable a more refined risk-benefit analysis.

Beyond biological efficacy, the clinical translation of noble gas-based neuroprotection faces substantial practical barriers that differ markedly across gases. For xenon, the most formidable obstacle is cost. Xenon constitutes only 0.087 ppm of atmospheric air and is obtained through fractional distillation of liquefied air, an expensive process that renders its price approximately US$10–20 per liter of gas—roughly 10–100 times more than conventional inhaled anesthetics. At a xenon price of US$10 per liter, the average cost for xenon anesthesia has been estimated at approximately US$65 for the first 15 min and about US$25 for each subsequent hour. For neuroprotective applications requiring prolonged administration (e.g., 24 h), the cumulative cost becomes prohibitive. To mitigate this, closed-circuit ventilation systems with gas recycling are essential; membrane-based xenon recovery technologies can achieve >99% recycling efficiency and reduce costs by up to four orders of magnitude, but such systems remain technically complex and not widely available in routine intensive care or emergency settings. A second major practical challenge is the oxygen-delivery conflict: effective neuroprotective concentrations of xenon in preclinical studies typically range from 50% to 75%, leaving only 25%–50% of the inspired gas mixture for oxygen. In patients with compromised pulmonary function, reduced cardiac output, or significant brain injury—precisely the populations most likely to benefit from neuroprotection—maintaining adequate oxygenation while delivering such high xenon concentrations becomes a critical safety concern. These combined factors—cost, recycling infrastructure, and oxygen delivery constraints—substantially limit xenon’s feasibility outside highly specialized centers with closed-circuit capabilities and rigorous ICU monitoring.

For argon, the translational landscape is fundamentally different. Argon is the most abundant noble gas in the atmosphere, accounting for approximately 0.93% of air composition, and its greater abundance translates to manufacturing costs of approximately US$2.71 per liter—an order of magnitude lower than xenon. Argon is also non-sedating, easy to transport, and can be delivered via inexpensive open-circuit ventilation systems without the need for closed-circuit recycling. These logistical advantages make argon a far more practical candidate for widespread clinical translation, and have led some investigators to argue that the time has come to consider Phase II trials in acute neurological injury. However, as discussed above, argon’s preclinical evidence base remains more heterogeneous than xenon’s, and its optimal dosing, timing, and interaction with concomitant therapies such as hypothermia are less well defined. Thus, while argon is substantially more accessible from a logistical and economic standpoint, its translational priority must be guided by further mechanistic and dose-finding studies rather than by cost alone.

For helium, a distinct interpretive challenge arises. In 2009, David et al. reported that 75% helium provided neuroprotection and improved neurological outcome in rats subjected to transient middle cerebral artery occlusion—but explicitly attributed this effect to helium-induced hypothermia “on account of its high specific heat as compared with air”. This finding raises a fundamental question: does helium possess intrinsic neuroprotective properties, or does its apparent benefit merely reflect the well-established neuroprotective effects of hypothermia, passively induced by helium’s physical properties? Subsequent studies have yielded conflicting results: some have reported helium-mediated protection under temperature-controlled conditions *in vitro*, suggesting possible direct effects, whereas others have found no benefit under normothermic conditions *in vivo*. The prevailing view in the field is that helium’s neuroprotective profile is highly condition-dependent and that its effects—when observed—may be largely explained by hypothermia rather than by specific molecular interactions.

## Clinical application scenarios and translational priorities

Future clinical applications of noble gases should be considered according to gas-specific evidence and practical feasibility rather than as a single uniform strategy. For xenon, the most realistic near-term clinical scenario may be post-cardiac-arrest brain injury, particularly as an adjunct to targeted temperature management (Laitio et al., 2016). In adult survivors of out-of-hospital cardiac arrest, inhaled xenon combined with mild therapeutic hypothermia for 24 h was feasible, achieved a median end-tidal xenon concentration of 47%, and did not cause unexpected serious adverse reactions or significant conduction, repolarization, or rhythm abnormalities (Arola et al., 2013). In a related randomized clinical study, xenon combined with hypothermia was associated with reduced MRI-based white matter injury, although improvement in long-term neurological outcome or survival has not yet been confirmed (Laitio et al., 2016). In ischemic stroke, delivery-based xenon strategies may be more feasible than conventional inhalation alone, as discussed below.

For argon, clinical translation should initially focus on feasibility and safety in highly controlled settings such as out-of-hospital cardiac arrest (OHCA) or cardiopulmonary resuscitation (Ristagno et al., 2025). Because preclinical findings are heterogeneous, especially with prolonged administration or combination with hypothermia, future argon studies should define the optimal timing, duration, concentration, and temperature-management protocol before efficacy trials are expanded. Argon should not yet be considered a clinically established neuroprotective gas.

For helium, future studies should prioritize preconditioning, pretreatment, or carefully timed adjunctive therapy rather than immediate stand-alone treatment. This recommendation is supported by neonatal rat studies showing that helium preconditioning can attenuate hypoxia-ischemia-induced injury and improve neurovascular repair. In adult rat focal ischemia, heliox was protective only when administered immediately after ischemia onset, supporting the need to define a strict treatment window (Pan et al., 2011). A small open-label feasibility study in patients with post-cardiac arrest syndrome treated with hypothermia showed that 3 h ventilation with a 1:1 helium/oxygen mixture was feasible and not associated with helium-related adverse events, but efficacy remains unproven (Brevoord et al., 2016). The Xe-He-37.5 mixture may also be explored as a xenon-sparing strategy, but its interaction with tissue plasminogen activator suggests that timing relative to thrombolytic therapy requires careful evaluation (David et al., 2017).

## Neurosurgical and delivery-related translational opportunities

Beyond acute brain injury models, neurosurgical anesthesia represents another potential translational context for xenon. Because xenon is already an approved anesthetic, its anesthetic use in brain surgery might theoretically coincide with possible neuroprotective effects. Several studies have shown that xenon may offer advantages over conventional anesthetics in terms of hemodynamic stability during neurosurgery (Rossaint et al., 2003). Law et al. (2016) also reported that patients receiving xenon anesthesia woke significantly faster than those receiving volatile anesthetics. In procedures such as awake craniotomy, rapid recovery from anesthesia may be clinically useful. In 2012, Kulikov et al. reported the first successful case of awake craniotomy under xenon anesthesia in a patient with preoperative speech deterioration and severe intracerebral hemorrhage (Kulikov et al., 2012). However, evidence in neurosurgical practice remains limited, and more clinical studies were needed. At the same time, xenon anesthesia also has important limitations. Postoperative nausea and vomiting (PONV) is a relevant complication, and after neurosurgery its incidence might be high (Latz et al., 2011). Law et al. found that PONV occurred more often in patients treated with xenon than in those treated with propofol (Law et al., 2016). In addition, xenon might raise concerns regarding intracranial pressure, which could make its use problematic in some patients with cerebral hemorrhage (Rylova et al., 2014). Achieving sufficiently high xenon concentrations might also be difficult in elderly patients or in those with respiratory disease because adequate oxygenation must be maintained. Therefore, perioperative anesthetic advantages should not be interpreted as proof of established neuroprotection in neurosurgery.

Because conventional gas administration is limited by low solubility, poor targeting, high gas consumption, and difficulty maintaining effective concentrations over time, novel delivery systems based on microbubbles and liposomes have emerged as potential strategies for more selective and efficient noble-gas delivery. In 2023, Peng et al. (2023) reported that xenon-loaded liposomes combined with recombinant tissue plasminogen activator (rt-PA) enhanced therapeutic efficacy, reduced neuronal cell death, and showed potential to mitigate hemorrhagic side effects associated with delayed rt-PA treatment. Such findings suggest that novel delivery systems may not only improve the biological efficiency of xenon itself, but may also expand the feasibility of using cheaper and more abundant noble gases by increasing their solubility in blood and tissue under normal-pressure conditions (Yin et al., 2022). In experimental stroke, xenon-containing echogenic liposomes administered within 5 h after stroke onset reduced infarct size, with an optimal dose range of 7–14 mg/kg, and this effect was associated with reduced ischemic neuronal cell death and activation of BDNF, Akt, and MAPK signaling. In a pig TBI model, ultrasound-triggered release of xenon-containing microbubbles at the carotid artery reduced perivascular inflammation and blood-brain barrier disruption, and endothelial cell experiments suggested preservation of the tight junction protein zonula occludens-1 (ZO-1). These findings suggest that localized xenon delivery may improve brain targeting, reduce systemic exposure, and lower gas consumption, although safety and reproducibility require further validation (Peng et al., 2013; Shin et al., 2023). Nevertheless, these technologies remain experimental, and further studies are required to evaluate their safety, reproducibility, cost-effectiveness, and applicability in clinically relevant settings.

## Risk mitigation in clinical application

Given the potential for xenon to elevate ICP, any future clinical application in acquired brain injury must incorporate predefined risk-mitigation protocols. First, patient selection is paramount. Xenon should be considered only in patients without established intracranial hypertension or significant mass effect. In patients with severe TBI or malignant stroke, xenon should be avoided or administered only as part of a carefully monitored clinical trial with explicit safety stopping rules. Second, concentration optimization should aim for the lowest effective neuroprotective concentration. Preclinical studies have reported efficacy at concentrations ranging from 30% to 75%, but higher concentrations increase the risk of ICP elevation. A pragmatic starting point for clinical investigation might be 30%–50% xenon, combined with ICP monitoring to assess individual tolerance. Third, concomitant ICP-lowering therapy should be considered, such as propofol sedation or targeted hyperventilation, to offset any xenon-induced ICP increase. Fourth, invasive ICP monitoring should be mandatory in any clinical study of xenon in brain-injured patients, with a predefined protocol for immediate xenon discontinuation if ICP exceeds a safety threshold (e.g., >20–25 mmHg) or shows a sustained rise. Fifth, novel delivery systems may offer a future pathway to circumvent the ICP issue. Xenon-loaded liposomes or microbubbles can achieve targeted brain delivery with potentially lower systemic concentrations, thereby reducing the risk of global vasodilation and ICP elevation. While these technologies remain experimental, they represent a promising direction for decoupling the neuroprotective effects of xenon from its systemic vascular and ICP-related side effects.

## Limitations and future directions

Several limitations should be considered when interpreting the current literature. First, most available evidence remains preclinical, and clinically established efficacy has not yet been demonstrated. Because this article is a narrative review rather than a systematic review or meta-analysis, no formal quantitative assessment of methodological quality, risk of bias, or publication bias was performed, and no pooled analysis was conducted to compare the neuroprotective effects of different noble gases. Therefore, the interpretation of the current evidence may be influenced by selection bias and the substantial heterogeneity of the included studies. Second, the literature search was limited to English-language publications, which may have introduced language bias and led to the omission of relevant studies published in other languages. This language restriction may have excluded relevant Chinese-language publications. A supplementary search of the China National Knowledge Infrastructure (CNKI) database identified predominantly preclinical mechanistic studies and narrative reviews on noble gas neuroprotection, but no Chinese national clinical guidelines or provincial multicenter trials specifically addressing noble gas therapy for acquired brain injury were found. Therefore, while the exclusion of Chinese-language literature represents a limitation, it is unlikely to have altered the main conclusions of this review. Nevertheless, future systematic reviews should consider incorporating both English and Chinese databases to ensure a more comprehensive synthesis. Third, marked heterogeneity among injury models makes direct comparison difficult. Stroke, post-cardiac-arrest hypoxic-ischemic brain injury, TBI, and perioperative neurosurgical injury differ substantially in pathophysiology, therapeutic window, and outcome assessment. In addition, treatment timing, exposure duration, gas concentration, temperature control, and selected endpoints varied considerably across studies, reducing standardization and limiting broad generalization. Fourth, the scope of this review was mainly restricted to acquired brain injury, and the potential roles of noble gases in other neurological disorders, such as neurodegenerative diseases, were not systematically discussed. Finally, clinical translation remains further restricted by practical issues, including xenon cost, oxygen-delivery requirements, gas-recycling systems, and technical limitations of gas delivery.

Future studies should therefore move from broad proof-of-concept experiments toward gas-specific, model-specific, and clinically oriented translational strategies. More studies in large animals and non-human primates are needed to narrow the translational gap between rodent experiments and clinical trials. Direct head-to-head comparisons among xenon, argon, and helium should be encouraged under standardized experimental conditions, including predefined gas concentration, exposure duration, treatment timing, temperature-management protocols, and long-term neurological outcome assessment. High-quality multicenter, randomized, double-blind, placebo-controlled clinical trials will be essential to evaluate neurological function, survival, quality of life, safety, and cost-effectiveness. Future studies should also systematically investigate combination strategies with established interventions, such as targeted temperature management, reperfusion therapy, thrombolysis, neurocritical care, and advanced delivery systems, while carefully defining the optimal treatment sequence and therapeutic window to avoid potential adverse interactions. In addition, precision medicine approaches incorporating imaging biomarkers, electrophysiological indicators, and circulating molecular biomarkers may help identify patients most likely to benefit from noble-gas-based therapy. Clinically feasible delivery systems, including liposomes, microbubbles, and nanobubbles, should also be further optimized to improve local gas delivery, reduce systemic exposure, lower gas consumption, and enhance translational feasibility.

## Conclusion

Noble gases have attracted increasing interest as potential neuroprotective agents in acquired brain injury, but their clinical role remains unproven. Among the gases reviewed, xenon currently has the strongest preclinical evidence and limited early clinical support, particularly in ischemic stroke, post-cardiac-arrest brain injury, and traumatic brain injury. Argon remains a promising but less established candidate, with heterogeneous and sometimes contradictory findings across experimental models. Helium is insufficiently studied, and its apparent biological effects appear to be highly dependent on temperature, pressure, concentration, timing, and treatment context. Overall, future studies should prioritize standardized experimental protocols, direct comparisons among gases, long-term neurological outcomes, safety assessment, cost-effectiveness analysis, and clinically feasible delivery strategies before noble gases can be considered established neuroprotective therapies.
